# The role of embodied scaffolding in revealing “enactive potentialities” in intergenerational science exploration

**DOI:** 10.1002/sce.21845

**Published:** 2023-11-06

**Authors:** Minna O. Nygren, Sara Price, Rhiannon Thomas Jha

**Affiliations:** ^1^ UCL Knowledge Lab University College London London UK

**Keywords:** adult, caregivers, child, gestures, movement, museums, video recording

## Abstract

Although adults are known to play an important role in young children's development, little work has focused on the enactive features of scaffolding in informal learning settings, and the embodied dynamics of intergenerational interaction. To address this gap, this paper undertakes a microinteractional analysis to examine intergenerational collaborative interaction in a science museum setting. The paper presents a fine‐grained moment‐by‐moment analysis of video‐recorded interaction of children and their adult carers around science‐themed objects. Taking an enactive cognition perspective, the analysis enables access to subtle shifts in interactants’ perception, action, gesture, and movement to examine how young children engage with exhibits, and the role adult action plays in supporting young children's engagement with exhibits and developing ideas about science. Our findings demonstrate that intergenerational “embodied scaffolding” is instrumental in making “enactive potentialities” in the environment more accessible for children, thus deepening and enriching children's engagement with science. Adult action is central to revealing scientific dimensions of objects’ interaction and relationships in ways that expose novel types of perception and action opportunities in shaping science experiences and meaning making. This has implications for science education practices since it foregrounds not only “doing” science, through active hands‐on activities, but also speaks to the interconnectedness between senses and the role of the body in thinking. Drawing on the findings, this paper also offers design implications for informal science learning environments.

## 1 INTRODUCTION

Although the benefits of family interaction and intergenerational construction of meaning about science have long been recognized (e.g., Eardley et al., 2018; Gutwill & Allen, 2012), research has primarily focused on sociocultural theories of collaboration and scaffolding strategies for supporting learning, often with a focus on verbal forms of interaction and verbal, material and social forms of scaffolding (e.g., Roberts & Lyons, 2017; Rojas‐Drummond et al., 2013). Alongside increasing interest in embodied approaches to cognition (e.g., Lübbert et al., 2021; Noë, 2006; O'Regan & Noë, 2001; Sheets‐Johnstone, 2011; Thompson & Varela, 2001; Varela et al., 2016), recent studies of family meaning making around science highlight the role of action and gesture in providing bodily forms of scaffolding to children's interaction with museum exhibits (e.g., Price et al., 2022; Tscholl & Lindgren, 2016). However, little work has focused on the moment‐to‐moment unfolding of embodied scaffolding and how aspects of science ideas can become differently sensorially accessible through action. The collaborative, social dimension of action is therefore less understood. This paper addresses this by undertaking a microinteractional analysis from an enactive cognition perspective to examine intergenerational collaborative interaction in a science museum setting with a focus on how adult's action supports a child's engagement with science‐themed objects and science ideas.

Enactive cognition argues for the importance of attending to perception and action when understanding sensorimotor skill development, where “action [is] a necessary prerequisite for perception” (Lübbert et al., 2021, p. 2). Thus, how we act matters, because it shapes what we can perceive. Alongside this, a substantial body of research shows the importance of the adult in supporting children's development in informal learning contexts (e.g., Povis & Crowley, 2015; Wood et al., 1976), specifically collaborative family interactions (e.g., Degotardi et al., 2019; Rojas‐Drummond et al., 2013; Wolf & Wood, 2012). Recognition of the importance of embodied forms of communication in scaffolding is increasing (e.g., Price et al., in press; Rowe & Kisiel, 2012). Indeed, O'Connor (2016, p. 13) notes the importance of understanding abstract concepts in “more embodied and experiential terms” and highlights how this may be achieved through “interaction with others who expand one's physical and cognitive potentialities.” Danish et al. (2020) also illustrate how interaction in group participatory simulations affords the “unfolding” of a science idea through collective, communicative action. However, less attention has been paid to the enactive features of (intergenerational) interaction and the relationship between the material environment and action in informal learning settings.

This paper extends our understanding of the enactive role of intergenerational interactions during young children's interaction with science in an informal setting. In particular, our analysis foregrounds sensorial and active experiencing, the role of action in embodied scaffolding, and how this shapes children's experience with science. We also present accounts of children re‐enacting dimensions of their interaction with science‐themed objects during a postinteraction interview, where nuances in children's gestural communication demonstrate the aspects children have gathered during their interaction. We speculate how moments of prior action and embodied scaffolding may have supported the development of these detailed accounts and how these relate to sensorial experiencing of science. Specifically, we focus on two dimensions of children's interaction with science ideas: (1) the sensorimotor unfolding of “enactive potentialities” (i.e., perceived opportunities in the environment to experience sensorimotor knowledge) linked to a science idea during intergenerational interaction, and (2) children's re‐enactment gestures which provide evidence for children putting sensorimotor knowledge into action in relation to science ideas. By so doing, we foreground science meaning making in the context of this paper as enactive processes, that is, the child's and adult's actions that build upon each other in their ongoing interaction, revealing different aspects of science and causality in the environment through action.

This analysis provides evidence for intergenerational enactive meaning making around science, as well as answering a call to analyze social interaction more broadly from an embodied cognition perspective (Ferreira, 2021). The paper makes three key contributions: (1) it demonstrates that adult‐child action exchanges with objects, as forms of “embodied scaffolding,” are instrumental in shaping the child's sensorimotor experience about science and understanding of causality; (2) it illustrates how a microinteractional analysis can provide insight into subtle shifts in what is observed and acted upon in the environment by interactants during intergenerational interaction and its role in enabling meaningful sensorimotor experiences with a science ideas; and (3) it informs design for science pedagogy and exhibition design from enactive cognition perspective.

## 2 BACKGROUND

### 2.1 Intergenerational interaction and embodied meaning making about science

The perceived value of intergenerational learning in providing meaningful and transformative learning is increasing (Fitzpatrick, 2019). In informal learning environments, such as museums (Ellenbogen et al., 2004), family interactions have been shown to support the development of inquiry skills (e.g., Gutwill & Allen, 2012) and experiencing of science through physical engagement (e.g., Lindgren et al., 2016). A commonly emphasized feature of family learning is the notion of collaboration (Ash et al., 2012; Ellenbogen et al; Heath et al., 2005); a “shared experience in which the discoveries of each individual are valued and important” (Shaffer, 2015, p. 113). The dynamics of these interactions contribute to children's meaning making through participatory sense‐making and active sensorimotor engagement (Degotardi et al., 2019; Price et al., 2021).

Intergenerational studies around science meaning making have typically focused on conversation (e.g., Jane & Robbins, 2007; Zimmermann et al., 2015). More recently, research is placing emphasis in the more embodied qualities of experiencing artefacts in museums (e.g., Chatterjee & Hannan, 2015; Thomas Jha et al., 2021; Price et al., 2022) and incorporating physicality in family learning about the natural world has been shown to shape intergenerational meaning making (Marin & Bang, 2018). Bodily actions can present as “concrete representations that serve as entry points for abstract ideas” (Shaffer, 2015, p. 113). However, the subtle ways in which learning is realized through different kinds of actions during social interaction with science exhibits has not yet received further attention (Shaffer, 2015). Although an embodied view on cognition argues that interaction with physical objects may support linking of sensorimotor processes to abstract concepts (e.g., Lakoff & Núñez, 2000), encouraging action on concrete objects has received criticism (Novack et al., 2014). One identified problem is that children focus on irrelevant details of objects or “perceptually rich symbols” (McNeil et al., 2009, p. 173), failing to generalize action with a single object (Uttal et al., 1997), and the process of interaction is prominent rather than the underlying concept. This paper addresses this by closely examining sensorimotor access to science ideas through analyzing subtle shifts in perception and action with science‐themed objects during intergenerational interaction.

### 2.2 Scaffolding and embodied scaffolding

The adults’ role during family visits to a museum has been seen as supportive to a child's exhibit interaction and learning (e.g., Ash et al., 2012; Eardley et al., 2018). This support has been characterized as a type of scaffolding. Drawing on the Vygotskian concept of “mediation” (e.g., Khojasteh et al., 2021; Sanford et al., 2007, p. 148), Wood et al. (1976) unpack the notion of “scaffolding” identifying the different processes through which social mediation can occur (e.g., Strömmer, 2016), whereby a more expert partner assists a novice in reaching better understanding or more independence in the learning process. A substantial body of research has studied the role of adults, more expert peers and technology in scaffolding children's learning, as well as sense‐making practices that speak to a socio‐constructive approach to learning. Within museum studies, the most significant finding is the importance of these family social interactions in scaffolding meaning making, particularly around interactive exhibits (e.g., Borun et al., 1997; Crowley & Callanan, 1997; McManus, 1987, 1992). Parents are shown to support learning and meaning‐making by using questions, giving explanations, modeling and sustaining attention and interest during interactions with exhibits (e.g., Leinhardt et al., 2002). Although the majority of work has focused on dialogic (verbal) forms of scaffolding (Rojas‐Drummond et al., 2013), more recent work highlights ways in which nonverbal forms of parental interaction in family museum visits play particular roles in “scaffolding.” For example, the role of touch as part of demonstration and exploration in socially mediated interactions (Rowe & Kisiel, 2012), or physical interaction to establish joint attention and interest (Zimmerman & McClain, 2016). Similarly, Price et al.'s, (2022) study of interactive tabletop interaction points to scaffolding activity primarily supporting engagement in the task of the exhibit rather than the scientific ideas themselves. Thus, little attention has been paid to how embodied forms of scaffolding of experience can support engagement with science ideas.

In the context of hands‐on learning, typical in museum settings, the value of examining detailed actions and action exchanges during intergenerational interaction is significant. This study addresses the research gap in “embodied scaffolding” in intergenerational interaction by foregrounding analysis on how adult “scaffolding” occurs through action with the interactive features of science‐themed objects, and how these actions can shape and support a child's subsequent interaction and experience with science. We use the term “embodied scaffolding” when discussing exchanges of actions between adults and children, and the sensorimotor and verbal strategies of support that adults offer children during the process of interaction.

### 2.3 Enactive approach to cognition

Understanding how young children come to comprehend their surrounding world and its phenomena is at the heart of human development research. Several learning and developmental frameworks, including Piaget's cognitive development theory (e.g., 1952), social learning theory (Bandura, 1986), information processing (e.g., Klahr & Wallace, 2021) “symbolic learning” (e.g., Stapleton & Stefaniak, 2019), “inquiry‐based learning” (e.g., MacLeod & Kilpatrick, 2001), and embodied learning (e.g., Abrahamson et al., 2020), have emphasized that drawing a learner's attention to salient features in the environment is an effective way to support learning. While these approaches also theorize action and experience, they are less concerned about the moment‐to‐moment sensorimotor interaction processes. The enactive theory proposes that perception and action are “fundamentally inseparable,” where “(1) perception consists of perceptually guided action and (2) cognitive structures emerge from the recurrent sensorimotor patterns that enable action to be perceptually guided” (Thompson & Varela, 2001, p. 173). This perspective foregrounds sensorial and active experiencing. It focuses on the relationship between perception and action and the subtleties of enactive potentialities in the environment; how things are perceived, how they are acted upon, and what this tells us about experience and knowledge in a situated context.

Empirical evidence in young children's meaning making about science has demonstrated how specific social and sensorimotor interaction processes may foster “sensorimotor contingencies” (Thomas Jha et al., 2021, p. 42; see also Gallagher & Lindgren, 2015; Gallagher, 2020; Hostetter & Alibali, 2008; O'Regan & Noë, 2001) that children can draw upon later when expressing their ideas (e.g., Callinan, 2014). This demonstrates a link between activity in the designed environment and the specific sensorimotor patterns that underpin conceptualizations of science topics embedded in that environment (Thomas Jha et al., 2021). “Cognition is best understood as enactive, as a form of situated practice rather than disembodied mentalizing” (Lübbert et al., 2021, p. 2). Enactive cognition thus emphasizes the process by which the person's own sensorimotor interaction supports the development of knowledge. This differs for example from social modeling, which emphasizes the role of mimicking, rather than the perception‐action connection in relation to (here) the science idea.

Noë's enactive approach places the process of perceiving as acting at the core of developing sensorimotor knowledge (2006, p. 9). Knowledge is thus conceptualized as an enactive process, where it is through the perceiver's skillful activity that they acquire content from experience in their environment (Noë, 2006, p. 3). For example, a child using a water pump in a museum exhibit focuses on the pumping action, but an adult's perceived skillful activity can support the child to make a link between their pumping action and the flow of water by placing a boat at the base of the flowing stream of water. Knowledge develops through us having experiences, which are necessary for us to understand our environment. Here, we define “experience” as the interactant possessing and making use of sensorimotor knowledge (Noë, 2006). To skillfully act in our environment to gain experiences (i.e., knowledge), we need to sensorially attend to the “actionable” elements of the environment (or enactive potentialities). In “sensing as acting” one can acquire sensorimotor knowledge. To have an experience with a science idea when interacting with science‐themed objects requires perceiving the enactive potentialities of those objects that expose that idea, or dimensions of it. However, objects can be observed or acted upon in a myriad of ways, and an interaction does not necessarily afford an experience with a specific science idea. A child visiting a museum with their parent may observe some of the qualities of water as they move their hand submerged at a water table, yet not experience, or make a link, with how their hand's movement creates a force that knocks over a boat at the other end of the table. Or they may mimic an adult's action to spin a wheel, but do not make a link between the nuances of that action and the force of the spin. Noë (2006) theorizes this kind of “void” of experience as “experiential blindness” (Noë, 2006, p. 3) to denote the difference between just perceiving and having an experience. There are different ways of experiencing a science idea sensorially (e.g., through vision, hearing, touch, movement, and so on). Thus, acknowledging the ocular centric nature of the term “blindness” (e.g., Adams & Aizawa, 2010; Gangopadhyay, 2010), the “blindness” in our work speaks to the interdependence of sensory perception and action in enactive cognition (e.g., Varela et al., 2016). Given that having an enactive experience is crucial to putting sensorimotor knowledge into use (Noë, 2006, p. 10), we explore the role of perceiving and acting in intergenerational scaffolding that supports the process of shifting from experiential blindness to experience with science.

Although Noë's treatment of enaction focuses on the individual, a social enactive approach highlights how our actions are embedded in and shaped by our social context (Gallagher, 2020). The notion of “interaction” and “joint action” are central to the social enactive approach (Gallagher, 2020, p. 113), where action becomes shaped by how each interactant individually takes part in an interaction situation. Interactants’ bodies coordinate in the environment through joint attention and joint actions that “lead to a buildup of meaningful action chains” (Gallagher, 2020, p. 113). Shared action brings meaning by enabling us “to share what is valuable and why it is valuable” (Nemirovsky et al., 2012, p. 293), and provides different emergent configurations that narrow and refine the field of perception. Given the intergenerational context of interaction in our study, this social enactive approach is a critical component of analysis.

This paper provides insight into how enactive potentialities become accessible during interaction between an adult and a child.

### 2.4 Research questions

1) How are enactive potentialities revealed through adults’ action, and taken up by children?
2) How does embodied scaffolding support young children's sensorimotor knowledge development with science ideas?

## 3 METHOD

**Figure 1:**
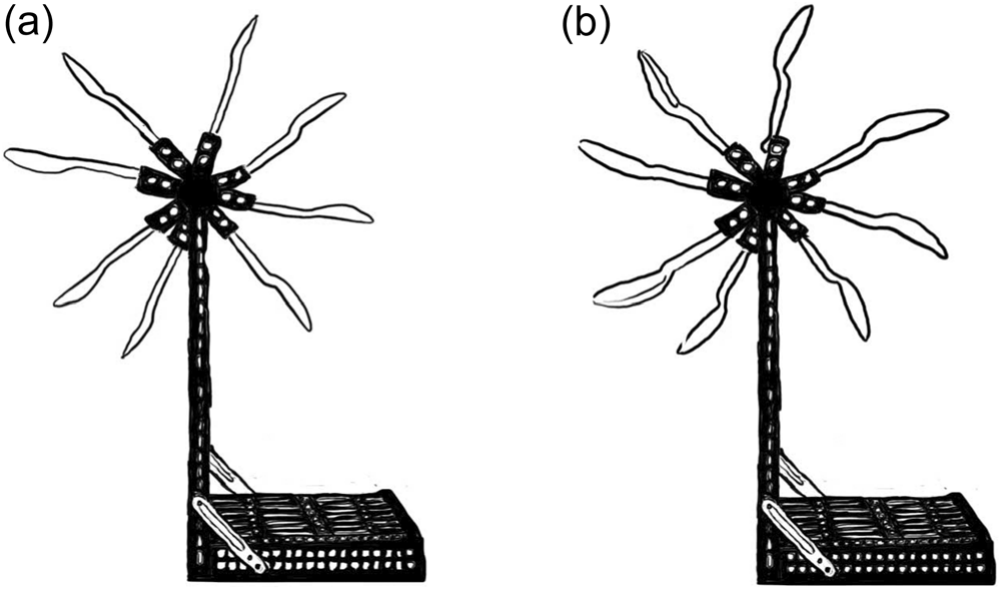
Illustrations of the water wheels designed for this study: (a) with teaspoons, (b) with tablespoons.

The study took place in the London Science Museum in a side room adjacent to an interactive early years’ science gallery (the Water Zone) where children and accompanying adults could interact with a water table and several purposely designed prototypical exhibit objects: two water wheels, a water tub, two water pumps and an Archimedes’ screw to explore the design of particular objects in supporting embodied forms of meaning making. In this analysis, we focus on intergenerational interaction with the two water wheels (Figure 1.).

### 3.1 Participants

Families with at least one child between the ages of 3 and 6 years accompanied by an adult were recruited on a voluntary basis at the entrance to the early years’ gallery by a researcher. They were informed that the study aimed to explore how families interact with purposely designed science artefacts to inform future exhibit design. Twenty‐seven children participated, across 20 family groups, consisting of seven pairs of siblings and 13 individual children (*M* = 4.81 years, SD = 1.36). Ethical approval was obtained from UCL ethics committee. All adults were given an information sheet and consent form, and the researcher talked through the process with the children with support from an age‐appropriate information sheet and gained their assent to take part. All participants were informed they could withdraw at any time. Names in this paper are pseudonymized for reporting purposes.

### 3.2 Materials

Two water wheels (Figure 1) were built with stainless‐steel flat bases that could rest steadily at the bottom of the water table. From their base, two vertical 20 cm beams held a rotating wheel attached at the top. On this rotating part, several transparent spoons were attached, acting as the water wheel “blades.” The water wheels were identical apart from the size of the blades; one made with teaspoons, the other one with tablespoons.

**Figure 2:**
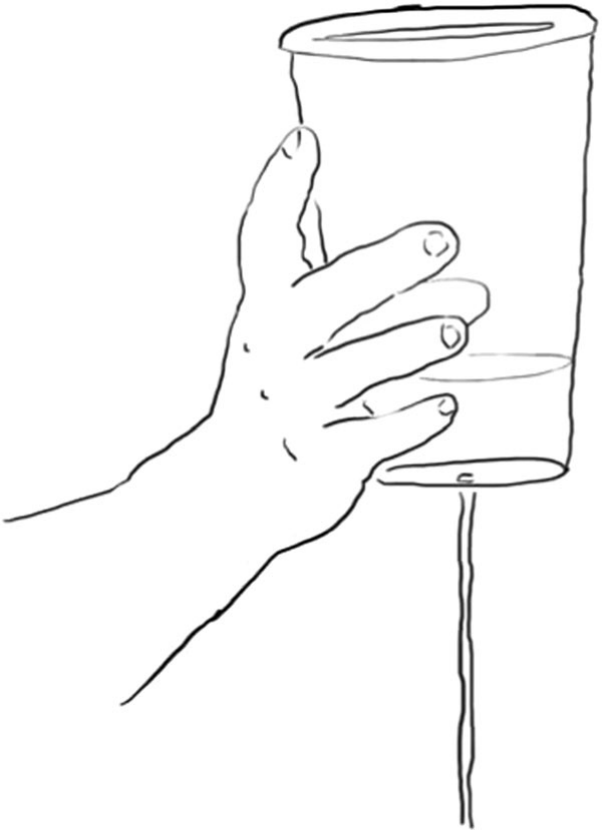
Illustration showing the transparent cup design with water pouring through the hole at the base while holding the cup up in the air.

Children were provided with two types of transparent cups which could be used to pour and create a flow of water. One cup had a hole at the base (Figure 2), filling it with water and holding it up resulted in a steady flow of water. The other cup had no hole allowing it to be filled and the water poured from the top of the cup by rotating one's hand holding it. The amount of water differently released from these cups could affect the rotation speed of the wheel, depending on how much water was in the cup, and the force of the pouring motion.

The water wheels were placed within a water table; a large 112 cm × 62 cm sized rectangular basin (TTS, n.d) made from translucent plastic and filled with about fifty litres of lukewarm water or up to 10 cm height. The basin's rim stood at 58 cm height off the floor. Next to the water table was one empty chair and a kneeling pad.

The scientific ideas available for exploration with the objects included changes in water flow, motion, volume, speed, and force. The rotating center of the wheel span the spoons attached to it in a circular manner whenever a force was exerted onto the spoons, for example by tapping a spoon with one's hand, or by pouring water on them from the cups. The speed of the spinning was affected by where the water was poured; pouring water directly onto the bowl of the spoon resulted in a faster spin. In addition, the different sized spoons affected the speed of the rotating motion; the larger blades captured more water exerting more force onto the spoon increasing the speed of rotation. As children moved the flow of water over the wheel the motion of the wheel changed, linking their motion, the flow of water and the motion of the wheel.

### 3.3 Procedure

Each family group participated separately in an interaction session with the water table and objects. Families were informed that the researchers were interested in how these objects supported family interaction and were asked to “interact as naturally as possible” with their child(ren). The researcher's role was to provide practical support and address any questions. The researcher provided the children with the cups and placed water wheels inside the water table, with their bases submerged, and the sides of the spoon blades facing the children. Families were given an open task: to explore how they could make the wheels spin and how they can “use water” to make them spin. They were asked, for example, “Can you see if you can make those wheels move around? What do you think affects the wheels’ spinning speed?” and “Do you notice any differences between the two wheels in the way that they look, or spin?.”

Families interacted with the wheels for ~5–10 min. Typically, the researcher brought the interaction to a close when they sensed the group's engagement had dipped. All interaction sessions were video recorded using one static camera, positioned to record all participants around the water table, yet to be as unobtrusive as possible. Although video recording potentially impacts interaction, video data is invaluable when exploring fine‐grained unfolding of interaction over time, “particularly where there is an interest in the use of gesture, bodily movement, interaction with objects and … L multimodal communication” (Jewitt, 2012, p.7). Heath et al. (2010) claim that “there is little empirical evidence that it [video] has transformed the ways in which participants accomplish actions” (p.49).

After interaction, families took part in a researcher led semi‐structured interview. While families were interviewed as a group, questions were directed to the children to encourage children's communication about their experience of their interaction. Families were seated with an open floor in front of them to enable children to use their bodies in communication. Interviews were structured around the objects presented at the water table. Children were shown photos of the objects and the interviewer used verbal prompts to encourage them to talk about their experiences with the wheels: “Remember this?” (showing the picture to the child), “How did you make the wheels spin around?,” “Where did you put the water?,” “And then what happened?,” “Do you know why?.” This design aimed to create the opportunity and space for children to reflect and recount their experiences with science‐themed objects and open new routes into accessing “embodied scaffolding” research, where verbal prompting used alongside images, can support children's communication and meaning making about their previous experience. Interviews lasted around 9 min and were video recorded; the camera being positioned to capture the group, their faces, gestures, and body movement.

### 3.4 Analytical process

Although interaction analysis has looked at details of movement in learning (e.g., Goodwin, 2018), family interactions (e.g., Shaby & Vedder‐Weiss, 2021; Zimmerman & McClain, 2016) and embodied experiences that foreground action and physicality have been explored in some CSCL work (e.g., Rosé et al., 2020; Stahl et al., 2014), they have not focused on the process of how enactive potentialities in the environment are discovered and acted upon by the interactants—how participants use action and enact sensorimotor knowledge in the environment that is aligned with a science idea. Core to our analysis was understanding how a child might come to have an experience with science idea sensorially, and how actions around this experience may be organized so that this experience is made accessible for the child.

Our approach to qualitative multimodal video analysis followed a method of “progressive refinement of hypotheses” (Engle et al., 2007) where a general theory‐driven question about intergenerational interaction with science‐themed objects was formed before data collection, and specific explanatory hypotheses were developed inductively across multiple viewings of the video data (e.g., Braun & Clarke, 2006, p. 84). The explanatory hypothesis initially focused on the notion of “action exchanges” between adults and children as shaping children's experience with science themed objects and science. For example, the adult picks up a cup, fills it with water, and carefully turns the cup on its side, letting some water pour out. The child, who has been observing the adult, picks up a cup, fills it with water, and initially turns it on its side, letting some water out, and but then extends the action by turning the cup upside down. Subsequently, more specific hypotheses about the role of adults’ actions in opening new interaction opportunities for the child were developed, with a focus on participants perceiving and acting on different opportunities in the environment. Given the focus on intergenerational interaction (a child interacting with their adult carer, be it their parent, nanny or grandparent), a subset of 12 videos were selected from the corpus of 19, where the child's carer—rather than the researcher—was the primary interactant with the child.

During the first round of analysis, two researchers viewed the videos twice over, making general descriptive notes about events where adult and child collaboratively interacted with the water wheels with a focus on participants’ actions. The whole team (three researchers) then discussed the data, shared and compared notes and developed an initial coding scheme. The primary focus at this stage was on observed “action exchanges” between the adult and child, and how these shaped the child's subsequent experience from an embodiment and science learning perspective.

During the second round of analysis one researcher developed the coding scheme by independently re‐viewing the data, detailing episodes of participatory sense‐making where children and carers attended and responded to each other's actions. Specifically, the researcher noted how participants’ actions with objects changed over the course of their interaction, and how adults’ actions shaped the child's actions and ideas about science. Another researcher viewed the data again to verify the coding, thus providing intercoder agreement. The research team then met to discuss the data, develop themes and compare notes. Any disagreements were resolved through team discussions. The units of analysis, or themes (Braun & Clarke, 2006, p. 88), were based on instances where an adult (a) physically changed the environment to enable a child to act in new ways with science, (b) actioned focal or hidden elements in the object in relation to science, or (c) modeled scientific relations through actions with object during collaborative interaction. During this stage, the researchers proposed theoretical conceptualizations for framing the selected data, initially drawing on embodied learning theory, refining this, as the analysis progressed, to (1) enactive cognition theory and notions of “perception” and “action,” “experience,” the “development of sensorimotor knowledge,” and “enactive potentialities,” and (2) developmental theory and notions of “scaffolding” and “embodied scaffolding,” as these concepts helped framing the data theoretically.

For the fourth round of analysis the video data was broken into shorter segments. At this stage, we adopted a microanalytical approach, drawing from (e.g., Goodwin, 1994, 2018). This approach focuses on analyzing communicative behaviors at a micro scale, and “rests on the notion that social interaction is organized through the observable actions and practices of participants, with which members of the collective make sense of each other's actions using a wide spectrum of embodied resources” (Katila & Raudaskoski, 2020, p. 448). Thus, it is interested in the process of interaction, foregrounding its multimodal nature (e.g., Price et al., 2022). Our approach also drew from Nemirovsky et al. (2012) analytical work that places moment‐to‐moment analysis of bodily engagement (gaze, gesture, action, movement, manipulation of objects) at the heart of meaning making about science concepts. Our microinteractional approach attended to frame‐by‐frame analysis of bodily action and gesture, alongside verbal communication to study how adults’ and children's interaction are shaped by each other's actions. We traced what enactive potentialities in the environment interactants “act upon” and how this affected meaning making about the science idea at each given moment. We examined the process of intergenerational interaction through analyzing the interactants’ re‐enactments, modeling actions, and verbal assertions about the objects, actions or science ideas. This approach was used to identify instances of intergenerational interaction where adult's action had an impact on the quality of the child's subsequent interaction with the objects and meaning making with and about science ideas (Derry et al., 2010).

The interview videos included instances of children recounting their interaction experiences drawing on body‐based resources including gesture and body action, suggesting a connection between instances of interaction with the objects and the child's meaning making about science.

Several terms around “movement” feature in this paper. By enaction we mean “the process of enacting sensorimotor knowledge” about a science concept relating to science‐themed objects. By “sensorimotor knowledge” from an enactivist perspective, we mean that the person “enacting” has an experience in which they connect an action through revealing an aspect about a science idea. We also use “action,” “gesture,” and “motion,” in our analysis when describing different modalities and ways of communicating, acting upon, and expressing about the environment in the material‐social context during interaction. We use “re‐enactment” when describing and discussing moments of enaction during interviews. This distinguishes between enaction with the objects during interaction and the interview during which children re‐enacted or in other words, referred back to their experience through embodied forms of communication. Thus, not all actions are enactions.

## 4 FINDINGS

For this paper, we draw on two examples from 11 instances across the data set, and which are representative of adult's embodied scaffolding activity. The examples consist of an interaction and an interview that demonstrate ways in which the adults participated in the interaction through bodily action and highlight the significance of these embodied interactions in supporting children's sensorimotor interaction with science‐themed objects and ideas, and children's subsequent communication of these ideas.

### 4.1 Example 1: Adult action, enactive potentialities, science experience

This example illustrates how adult action functions as a catalyst for a child to enact previously hidden elements within the environment and enact sensorimotor knowledge about a scientific concept in their own way. It draws on an interaction and interview between two siblings Matthew (6 years) and Oliver (4 years), their grandfather and the researcher to illustrate, from an enactivist perspective, how the grandfather's embodied scaffolding guides Matthew's science experience. Here we focus on three consecutive episodes of interaction between Matthew and his grandfather, followed by Matthew's recounting of his experience in his interview. The first episode shows the enactive potentialities in relation to sensorimotor interaction with the science‐themed objects Matthew enacts on the environment. In the second episode Grandfather joins the interaction to make visible to Matthew an aspect about using the wheels that Matthew had not perceived as influencing the wheel's spinning speed. In the final episode, Matthew builds on this to explore different actions with the wheels. Matthew finishes with a new inference about the effect his own action has on the world. These excerpts provide evidence of Matthew having experiences with novel enactive potentialities with the objects and science concepts that are revealed by the grandfather's enaction on the objects. The interaction episodes are followed by two interview excerpts, which present the ways in which Matthew recounted his experience with science ideas, and the details that his gestural communication reveals about these experiences that provide evidence of his developing sensorimotor experience with the water wheels.

#### 4.1.1 Interaction episode 1: Matthew makes an inference about his own enaction

**Figure 3:**
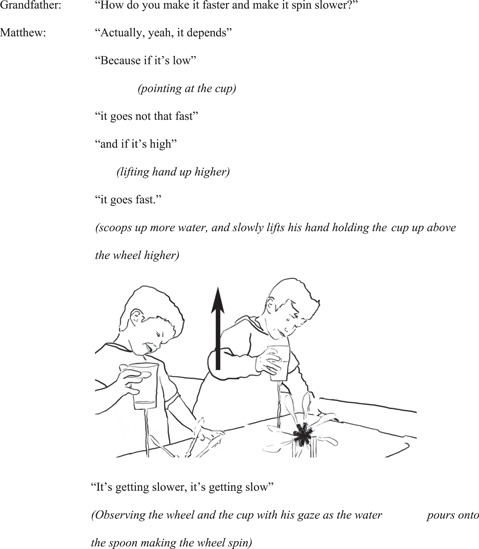
Interaction episode 1. Matthew lifts his cup up above the wheel.

After initial playful exploration of the wheels, the researcher asks Matthew and Oliver how they might make the wheels spin faster. The grandfather repeats the question to the children. Matthew, holding the cup with water running out from the base of it above the wheel, explains his idea about what affects the wheel's spinning speed (Figure 3).

At the beginning of the episode, Matthew points at the cup and verbally describes how when “it” is low the wheel does not go fast, and when “it” is higher, the wheel goes faster. At the end of the episode, he demonstrates how the wheel spins slower as he brings the cup lower. However, he also lifts his hand holding the cup up as he does this.

#### 4.1.2 Interaction episode 2: The grandfather brings new enactive potentialities

**Figure 4:**
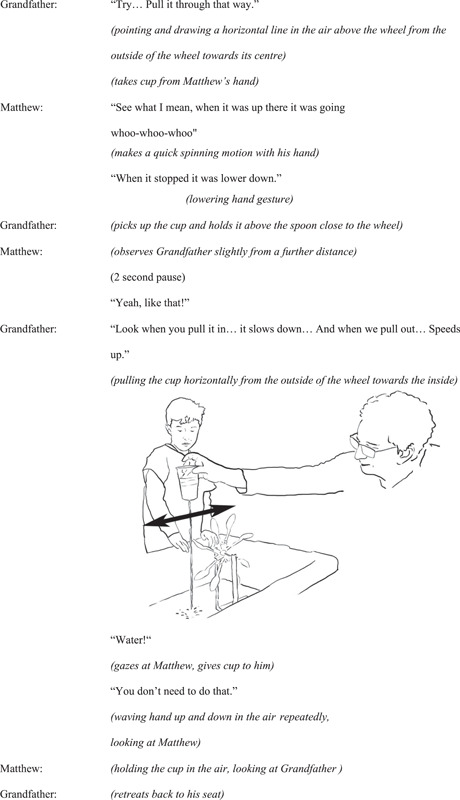
Interaction episode 2. Grandfather demonstrates how this movement affects spinning speed.

Once Matthew has demonstrated how his enaction (perceived action‐effect relationship) affects the wheel spinning speed, the grandfather (who has been observing the exploration between the two siblings) engages, through action and speech, guiding Matthew by using a new enaction with the cup; moving the cup horizontally from the outside of the wheel to directly over the spoons, thus maintaining the cup at the same height, which offsets Matthew's up‐down action. He finishes by re‐enacting Matthew's previous action, verbally emphasizing how this action does not affect the wheel spin speed, prompting Matthew to try this action to change the speed of the wheel. The grandfather then retreats to his seat (Figure 4).

With his actions and verbal assertions, the grandfather draws Matthew's attention towards enactive potentialities that make the underlying science idea more explicit and salient. The grandfather's demonstration seeks to counter what has been focal to Matthew's perception and action so far and highlight the enactive potentialities linked to the science idea (see Interaction episode 1). Through his enaction, the grandfather demonstrates a new action that reflects the cause and effect of positioning of the cup that makes the wheel spin or not. In this way, the grandfather is making the action‐speed of the wheel connection focal and more precise. In addition, by exclaiming “water,” he emphasizes the water's positioning on the spoon as a key component to the wheel spinning. Finally, the grandfather demonstrates how it is not necessary to move the cup up and down for the speed to be affected by re‐enacting Matthew's earlier lifting action.

This episode speaks to the idea that objects have different properties that lend themselves to science exploration in different ways. Some, such as the water falling through the hole, are merely observable. However, other actions, such as moving the cup in different ways exposes a specific cause‐and‐effect relationship relating to the spinning of the wheel—when the water falling from the cup is anywhere else except right on the bowl of the spoon, the wheel's speed is reduced.

#### 4.1.3 Interaction episode 3: Matthew has an experience with the underlying science idea

**Figure 5:**
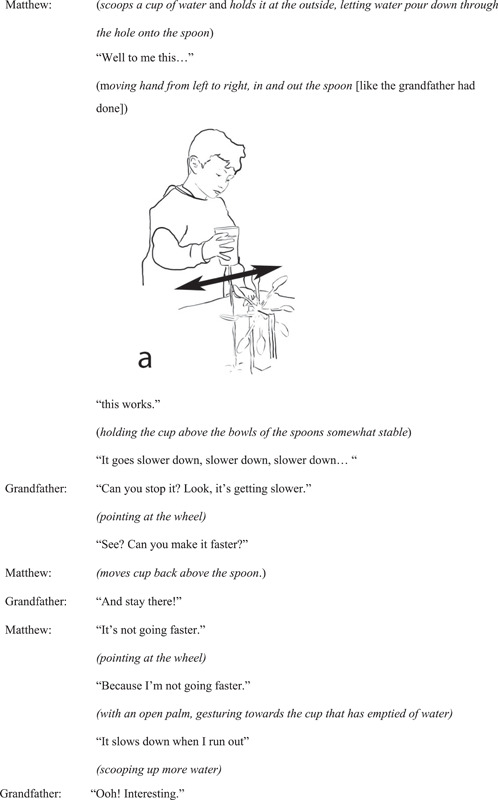
Interaction episode 3 demonstrating moments where Matthew makes two distinct inferences about his experience with the wheel and what affected the wheels’ spinning speed.

Actions from the grandfather, from the above episode, lead Matthew to re‐focus his attention on his own action and as he further explores using his grandfather's action and his own, he develops detailed sensorimotor knowledge about the phenomenon; how he can make the wheel spin faster or slower (Figure 5). Thus, his action becomes an enaction; he is developing an enactive account of the sensorimotor knowledge.

Here, Matthew has an experience with science through enacting on the objects in a way that makes a cause‐and‐effect relationship more explicit. After having observed his grandfather's action on the objects, he now puts this sensorimotor experience into use through enacting on the cups himself. This enaction marks a shift in Matthew's exploration as he is now connecting his actions with the objects to underlying cause‐and‐effect relationships between the position of the cup pouring water on the spoons and the speed of the wheels. Matthew has had an “experience” with enactive potentialities aligned with science ideas and has moved away from “experiential blindness” in an enactivist sense. His grandfather's enaction has thus functioned as a form of “embodied scaffolding” that has revealed enactive potentialities making the science ideas more explicit.

##### Interview

With two excerpts from the interview featuring Matthew's recounting of his experience through re‐enactments, we demonstrate that his experience with the objects foregrounded the interconnectedness between water flowing onto the spoon, and the speed of the wheel.

#### 4.1.4 Interview excerpt 1: Matthew re‐enacts the motion and shape of water pouring out through the hole in the cup as the element that affected the wheels’ spinning

**Figure 6:**
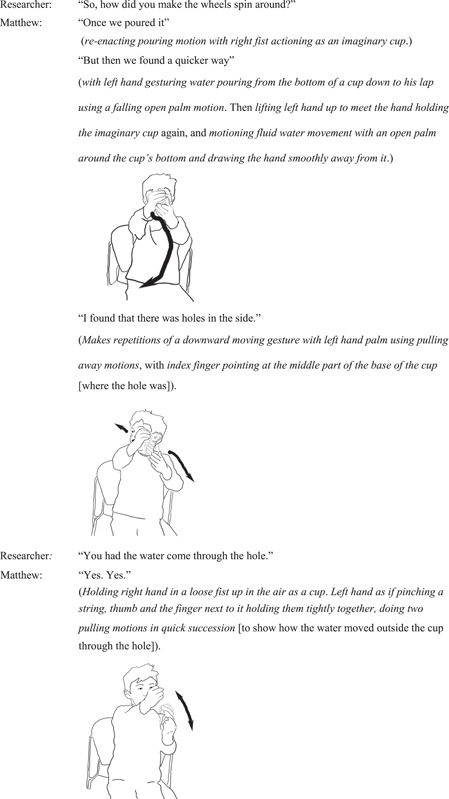
Interview episode 1: Matthew re‐enacts continuous different qualities of the flow of water.

Matthew responds to the researcher's question about how he made the wheels spin around by re‐enacting with his hands the details of the qualities of water flowing out through the hole in the cup when holding the cup in the air in a static position. With three re‐enactments, he demonstrates an awareness of the smoothness of water as it moves, the shape of water flowing out as a string, and its falling speed. Through re‐enactments, he communicates details about water flow, and its relationship to the wheel spinning (Figure 6).

During his re‐enactment of the cup and water flowing out through the hole at its base, Matthew is engaged in showing small dynamic details about his experience. The way in which he pinches his fingers tightly together drawing lines in the air evokes the shape of a thin stream of water flowing out through the base of the cup; the fluidity and smoothness of the turning and descending motions he makes with his relaxed palm from the base of the imaginary cup evoke many aspects of water as an element; its shape, movement and speed—all captured in Matthew's communication.

#### 4.1.5 Interview episode 2: Matthew re‐enacts the placement of water onto the bowl of the spoon

**Figure 7:**
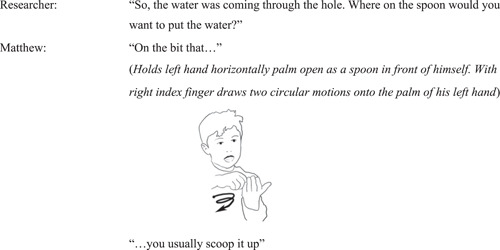
Interview episode 2. Matthew re‐enacts the water falling onto the middle part of the spoon.

In answer to the researcher's question about the placement of water on the spoon, Matthew re‐enacts the water pouring down onto the onto the bowl of the spoon: with his left hand, he creates a bowl shape facing upwards, and his index finger from his right hand moving onto the middle of it to denote how a string of water arrives onto the specific part of the spoon from above. He then repeats a circular motion with his finger to convey how a stream of water moves on the surface of the spoon as it hits it (Figure 7).

Matthew's re‐enactment gives evidence of his understanding of the necessity of the water to hit the “right spot” on the spoon to make the wheel spin. The precision of his re‐enactment indicates that he has “experienced” water flowing onto the specific spot on the spoon to gain a desired effect, for the wheel to spin optimally and fast.

These two excerpts show that Matthew's gestural re‐enactments convey enactive experiences he had had during the interaction. These gestural depictions contain detailed observations about water shape, speed, and movement that he expresses gesturally and verbally and reflects his experience with his grandfather which focused on the positioning of the flow of water. Thus, Matthew's sensorimotor knowledge is shaped by his experience enabled by Grandfather's embodied scaffolding with features or aspects of the environment relevant to the science idea becoming focal within the landscape of enactive potentialities.

### 4.2 Example 2: Benjamin and his grandmother: A social enactive encounter

Our second example, with two siblings; Benjamin (4 years) and William (6 years), their grandmother and the researcher, illustrates how forms of “joint actions” create “meaningful action chains” (Gallagher, 2020, p. 112.) with the water wheel and the science idea. We detail three episodes to demonstrate how grandmother co‐ordinates Benjamin's attention to lead their engagement in joint action, then undertake several intentional joint actions culminating in a mutual goal of making the wheel spin fast, with Benjamin leading joint action exploration on the other wheel. These episodes demonstrate how joint “embodied” action contributes to experiencing enactive potentialities with science objects and development of sensorimotor knowledge during intergenerational interaction.

#### 4.2.1 Interaction episode 1: Benjamin re‐engages with the wheel after grandmother proposes a new action

**Figure 8:**
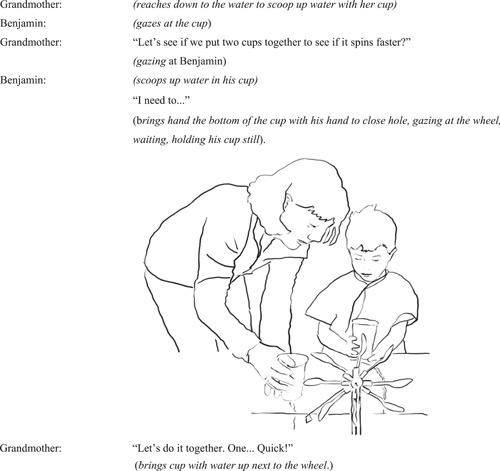
Interaction episode 1: Grandmother and Benjamin ready their cups with water.

In this episode, grandmother and Benjamin co‐ordinate intercorporeally (Gallagher, 2020, p. 109) in a number of ways—using action and speech—that demonstrate shared intention to act on the object; here, to try to make the wheel spin faster (Figure 8).

Grandmother moves closer to Benjamin allowing the couple to jointly attend to the wheel, which becomes a shared object between them. The placing of her hand on the cup whilst it is still sitting in the water captures Benjamin's attention. Gazing at Benjamin, the grandmother directs further prompts for a new action through speech, encouraging him to take part in a joint action. Through mentioning “spinning faster” she also sets a goal for the joint action, enhancing intentionality that makes “motives and reasons for acting more explicit” (Gallagher, 2020, p. 100). Thus, this moment, where enactive potentialities are just “budding” or not yet acted upon, becomes a space for initiating the joint action. Both interactants seem to have a “perceptual sense” about the presence of this enactive opportunity (Noë, 2006, p. 216); their scooping up of the water conveys this intention. Benjamin, who already “owns” the sensorimotor knowledge from having poured water onto the wheel to make it spin, becomes differently engaged when presented with a new enaction; a new sensorimotor opportunity in relation to science.

This experience, where the grandmother played an important role as a collaborator and a facilitator, functioned as an “embodied scaffold” that renewed the child's interest in exploration and further testing during the interaction.

#### 4.2.2 Interaction episode 2. Benjamin and grandmother pour water onto the wheel together

**Figure 9:**
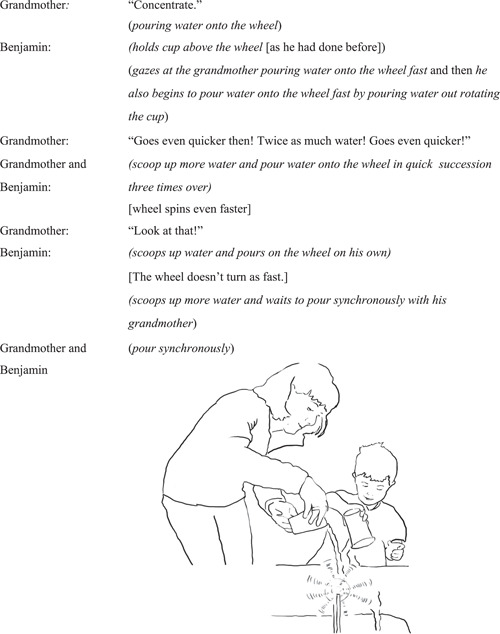
Interaction episode 2. Benjamin and grandmother pour water onto the wheel simultaneously.

In this episode, grandmother and Benjamin engage in an interaction that constitutes a series of iterations of the pouring action: joint actions, intentional actions that are generated mutually and individual responses to these. Through these “action exchanges,” the couple builds, action‐by‐action, a “loop of action iterations” with the theme of pouring water, where the shared goal of making the wheel spin very fast is reached through both pouring much water onto the wheel at the same time (Figure 9).

Engaging in joint action does not require a great deal of verbal communication. Joint action is maintained through bodily and communicative movements and leads to a complex joint action that is led by continued movements and repetitions of these movements (interactants returning to fill the cups with water) and becomes a moment where the enactive potentialities are fully experienced by both interactants simultaneously. Although these joint actions are pragmatic and goal‐oriented (to make the wheel spin faster by pouring more water onto it together), there is also an attentiveness to the quality of the “goal as interaction” that emerges over time in relation to the different phases involved in the pouring techniques. As the couple keeps pouring water on the wheel, there are times when their joint actions get “out of sync” and the water wheel does not spin as fast. Towards the end, this is compensated for by Benjamin waiting for his grandmother to scoop the water up, and only when she brings her cup and is ready to pour, does he pour onto the wheel at the same time. This relies on him having had an earlier experience at the beginning of the joint action of the wheel spinning faster when two persons pour water on the wheel at the same time.

In encouraging Benjamin to jointly interact with her to make the wheel spin very fast, grandmother brings the possibility of augmenting the action and the maximum sensorial effect; two people pouring water at the same time, results in a larger sensorial effect as the wheel spins faster than when Benjamin alone was pouring water onto the wheel. Here, as with the previous illustrative example, it is evident that the adult brings their own set of sensorimotor knowledge and experience to the event (Noë, 2006). Here, it becomes a creative action event where both adult and child contribute to the same action, and powerful collaborative pouring of water on the wheels to make the wheel spin faster than previously.

#### 4.2.3 Interaction episode 3. Benjamin initiates repeat of interaction on the other wheel

**Figure 10:**
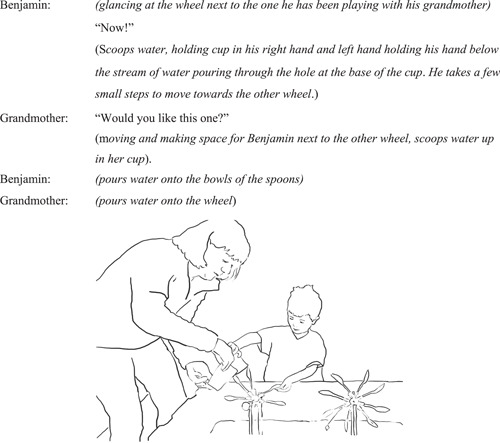
Interaction episode 3. Benjamin leads the interaction on the other wheel.

Benjamin's action experience with his grandmother seemed to be a meaningful experience for him since he then moves to the other water wheel to continue his exploration, encouraging his grandmother to join him in recreating the same joint experience on that wheel (Figure 10).

As Benjamin leads the couple's move onto the next wheel, Benjamin turns towards the other wheel, and his brief shout “Now!,” convey an action intentionality that his grandmother responds to by moving her body to make space for her grandson to act on the other wheel. Pouring more water and having the experience of the wheel spinning faster through two persons joining in the same action, is realizing an enactive potentiality that was not there before grandmother's embodied scaffold.

These episodes raise the interesting notion of enactive “blind spots”—the hidden potentialities in our environment, and how some of these experiences only become available through joint action. This example also talks about experience in the enactive sense; however, it focuses on the social aspects of a young child experiencing science with objects, together with another through enaction. Action enabled Benjamin to access new experiential qualities of science ideas that “augmented” previously experienced phenomenal content and prompted further exploration with a sensorimotor knowledge experienced together with another.

##### Interview

During the postinteraction interview, Benjamin enacted detailed phenomenal features of the experience giving evidence of how Benjamin's sensorimotor experience with the water wheel shaped his thinking about water, and the knowledge he developed during interaction. Specifically, he recounts through enaction different aspects of the scientific concept that relate to ideas of force: the dynamics of water's movement and weight as it falls on the spoon and how this in turn affects the spoon's movement. The details Benjamin re‐enacts during the interview have congruency with the science idea available in the experience with the object and demonstrate a deeper understanding of causality.

#### 4.2.4 Interview episode 1: Benjamin manually re‐enacts water pushing a spoon down

**Figure 11:**
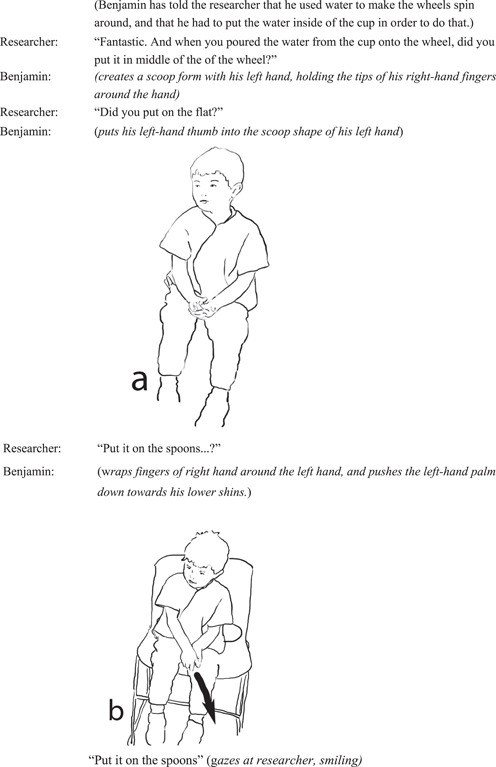
Interview episode 1. Benjamin re‐enacts shape of bowl of the spoon and water falling.

Through re‐enactment, Benjamin goes beyond describing the physical qualities of the spoon as an object, to convey beyond verbal description and speech modality, to show the pushing of the spoon by the water, thus conveying force of the water, and a causal effect of the water hitting the spoon to make the wheel spin (Figure 11).

The notion of force is focal in Benjamin's gestural recounting of putting water onto the spoon. This is conveyed by his pushing of his hand down towards the ground, almost in an exaggerated manner. The pushing motion conveys force of the water, and the effect this has on the spoon, which lowers down as a result.

From an enactivist perspective, this re‐enactment indicates that Benjamin's experience with the objects and the underlying science idea through the experienced event “water falls onto spoon—spoon goes down” has been qualitatively focal to him. Through the effort he is exerting onto the spoon during his re‐enactment, he conveys an important detail about the science concept related to force. This quality of action is reminiscent of the quality present in the interaction between Benjamin and his grandmother, when they together, poured water onto the spoons creating a rich sensorial experience that may not have been available for a single person to complete on their own, and possibly without an adult. Benjamin's re‐enactment in this interview excerpt shows the amount of detail and nuances he has picked up from his experience with the water wheels, and the cause‐and‐effect relationship between water and the spoons. This episode gives further evidence about young children's multimodal communication, and the importance of attending to children's re‐enactive practices as they can communicate a wide range of details drawing from their body‐based resources evoking detailed events and relationships about their world and experiences.

#### 4.2.5 Interview episode 2: Benjamin re‐enacts the wheel moving and the water falling

**Figure 12:**
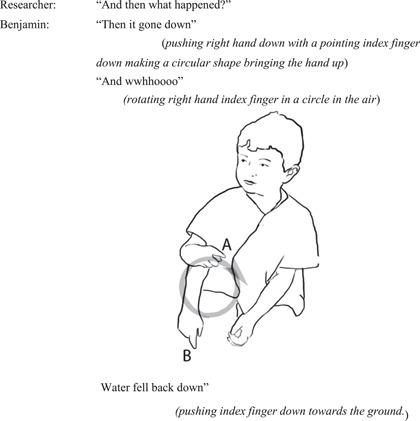
Interview episode 2. Benjamin re‐enacts wheel's spinning and direction of water falling.

When the researcher asked Benjamin about what happened next, he uses a circular pointing action to re‐enact the wheel's spin towards the ground and then back up again (Figure 12).

Benjamin re‐enacts the turning of the wheel first on its own, then adding his own vocalization to the movement. His right‐hand finger first rotates and traces the circular motion in the air, conveying the direction of the spinning of the wheel. Next, he repeats the rotating action this time he adds a vocalization to his action. In his final re‐enaction, he moves on to conveying the water falling back down into the water table. The index finger, still held up in the air, now becomes the water, and he pushes it down to show how the water “went down.” With this action, he is making the separation between two interconnected yet distinct (individual) events: the wheel turning, and the water falling. His description of the two events conveys a detailed understanding of the different parts involved in the process of the wheel spinning, and the water's role in this. To this open‐ended question about what happened, he is describing two focal elements and their enactive potentialities and sharing his experience through a combination of verbal and gestural retelling.

## 5 DISCUSSION

This paper makes three overlapping contributions. First, it brings focus to the role of action, as an embodied form of scaffolding through revealing enactive potentialities in intergenerational interaction on young children's sensorimotor knowledge development; second, it illustrates how a microinteractional approach to analysis using an enactive cognition lens brings detailed insight into the role of social action experiences in supporting science learning through embodied scaffolding of sensorimotor experience; and third, it lays out implications for science pedagogy and informal science learning design from an enactive perspective.

### 5.1 Intergenerational action: Revealing enactive potentialities through embodied scaffolding

Our analysis has demonstrated that adult action can function as a form of “embodied scaffolding” during intergenerational interaction. The child is not merely mimicking, or re‐enacting, what the grandfather has demonstrated. Rather, he is actively involved, attentive and focused in making meaning through the new action, observing dimensions of the effects of this new action, as well as verbally expressing his experience. Specifically, we highlight that adult enaction can draw out such enactive potentialities and reveal them to a child.

Adults employed different enactive strategies. Both the grandfather and grandmother engaged in scaffolding practices that drew on the enactive potentialities with science ideas and objects in the environment. In this way, each adult capitalized on the use of objects as “affordances” for embodied participation to broaden the experience of science for the children. Both grandparents scaffolded using enaction (using their sensorimotor knowledge about a science idea related to the objects) to make the relationship between an action and the science idea more noticeable, perceivable and accessible for the children. In this way, they foreground embodied and experiential details relevant to revealing important relationships between the objects, action and the science idea, support linking of sensorimotor processes to abstract concepts (e.g., vLakoff & Núñez, 2000).

The findings suggest that such embodied scaffolding supports young children to have what Noë calls “an experience.” For example, evidence from the data suggests that Matthew has had “an experience”; he perceived, and put into action, material and enactive properties of the cause‐and‐effect relationships available in the interaction. Although interaction with his grandfather was instrumental in Matthew's engaging with these enactive potentialities, the interview shows Matthew recounting his experience with the water wheel, and the detailed aspects he has noticed in the environment and interacted with; the importance of the water hitting the center of the spoon, and how the water moved on the bowl of the spoon once it hit it, as well as the different shapes the flow of water took when it was pouring out through the base of the cup. In his interview answers, he re‐enacts these elements that suggest he was engaged with several enactive potentialities during his experience in relation to the qualities of water.

Although speech may be part of the interaction, it is the quality of the action which is key to the way the science processes are revealed. We foreground the notion of “enactive” in “embodied scaffolding,” as it highlights the perception–action link—what is noticed in the environment, and what is acted upon, how it is done, and the outcome for the child's experience. Thus, the findings provide insight into how enactive potentialities are revealed during sensorimotor interaction between an adult and a child, thus shifting from what Noë calls “experiential blindness” to “experience” (2006, p. 3). At the same time, it reveals how the dynamics of interaction shape and bring about meaningful action chains through participants’ engagement in “joint action” (Gallagher, 2020, p. 113). In so doing, the findings broaden our understanding of scaffolding amidst the more well‐known modalities of support including language, social actors, and technologies (e.g., Khojasteh et al., 2021).

Objects for learning are often designed to be used in prescribed ways to afford learning experiences. However, this analysis shows that there are multiple ways to act upon objects, and although this is not surprising per se, it demonstrates the challenge of making abstract concepts “accessible” through physical objects directly and precisely. A detailed fine‐grained analysis demonstrates the types of interactions that can happen when an object is shared between two participants; an adult and a child. In this way, we show that objects can be used as scaffolds through sensorimotor interaction, to bring relevant scientific details into focus linking the underlying concept with action; the objects become part of meaning making and an exchange of ideas around more abstract science ideas. From a focus on intergenerational interaction and participatory sensemaking, we build upon the emerging work in embodied meaning making and the supportive evidence for what has been called action‐based learning (Novack et al., 2014) from an enactive and sensorimotor perspective. This contributes to our understanding of the importance of intergenerational (or other collaborative) sensorimotor interaction with objects. Specifically, we can see how, what has often been described as a pitfall of action‐based learning, where children may be prone to focus on “irrelevant” details of objects (McNeil et al., 2009), the attentive and “enactive” adult can scaffold by bringing in the enactive details of the objects, capitalizing on their sensorimotor qualities as abstractions of science concepts.

The findings also speak to the notion of “social sensorimotor contingencies” (Lübbert et al., 2021) in the way that the science is unveiled through a process of interactants, through enaction, both emerging from the interaction as deploying “learned action‐effect contingencies” from a social perspective. In the case of Matthew, it centered around a dialog around what in the environment affects the wheel's spinning speed, resulting in a “dialogic action response” to explore what affects the wheel's spinning speed. In the case of Benjamin, the learned action‐effect contingency from a social perspective is how the grandmother and grandson socially orient towards togetherness and play, as their pouring of the water becomes a moment of joyful joint action to pour water together and make the wheel spin very fast. Museums, while presenting interactive galleries to explore science through embodied ways, are, of course, also spaces that nurture dialog and social exchange, and are thus optimally placed to foster social action experiences with science.

### 5.2 Methodological contribution

Our analysis responds to a recent call for new methodological approaches that analyze collaborative and embodied interactions in children's learning from an embodied cognition theoretical perspective (Ferreira, 2021). While embodied exploring of science ideas through intergenerational interaction aligns with other research (e.g., Marin & Bang, 2018) our approach highlights the opportunities for attending to the subtle nuances of interaction and shifts in perception and action around science ideas. It extends our understanding of embodied forms of scaffolding (e.g., Danish et al., 2020; Rosé et al., 2020) through a focus on the processes of intergenerational interaction and the moment‐to‐moment shifts in interaction.

A focus on the process of meaning making through a microinteractional description of sensorimotor exploration and communication (involving body action, gesture, gaze and body positioning) and how these change over time enables insight into ways in which “enactive potentialities” are revealed. It demonstrates how action functions in drawing attention to specific features of an object, not just in terms of how they can be used, but also, how they clarify or expose another perspective on, for example, a cause‐and‐effect relationship as in the case of Matthew and his grandfather. In the case of Benjamin and his grandmother, moment‐to‐moment unfolding of interaction revealed how different enactive potentialities are revealed through joint action, and the associated qualitative shifts that happen in terms of sensorial feedback in an environment. The examples demonstrate how it is during the adults’ noticing and being attentive to the children's enactive practices in informal science learning spaces, during what often seem just short instances, where we discover how the child perceives the world and cause‐and‐effect relationships.

Taking a similar analytical approach to post interaction interview data, our examples demonstrate the importance of attending to children's enactive practices; how children's re‐enactions draw on body‐based resources, or their sensorimotor knowledge, to communicate a wide range of details about the events they have experienced, and the complex enactive material relationships within these experiences. Enabling children to express themselves multimodally opens new doors into how we, as adults, understand children's conceptualizations about the natural world and the scientific phenomena as they develop their understanding through sensorimotor interaction in different situations. Our findings thus contribute to existing research into children's gesturing about science and their narrative practices (e.g., Callinan, 2014; Thomas Jha et al., 2021; Price et al., in press), and through our examples speak to the social and intergenerational interaction as supporting sensorimotor knowledge development from an enactive perspective. This evidence points to how children can have what Noë calls “an experience.”

### 5.3 Implications for science exhibit design

These findings point to three design implications for science pedagogy and exhibit design: (1) the importance of foregrounding the perception and action link in experiencing science, (2) science exhibit designers or educators drawing on a social sensorimotor perspective, and (3) children being invited to re‐enact aspects of their experiences after interaction with science‐themed activities. In the following, each of the three implications are discussed.

First, the findings highlight the importance of design dimensions that foreground the perception and action link in experiencing and learning about science. In particular, they point to opportunities for designers to explore what elements of science are accessible through sensorial means in objects and how different types of actions, or sensorimotoric engagement, can foreground specific aspects of science ideas. The findings demonstrate the importance of adults guiding children's science exploration through “active” action demonstrations, thus opening new “enactive potentialities” or ways for children to explore and access science ideas. For example, exhibits could have illustrative “re‐enactment signs” that demonstrate not only how to “use” an exhibit, that is, how it functions, but beyond this, incorporating and presenting subtle action exploration chains that make different causality relationships more explicit. Drawing from our first example, a “sign” with the cups and wheels could include suggestions for actions such as the one first presented by Matthew—to hold the cup above the spoons and lift the cup up slowly—to see if it affects the wheel's spinning speed. Next to it, another action can be given, such as that demonstrated by the grandfather, to lend another perspective to the experience and science idea. These “signs” could be used not only with physical objects, but also in other spaces, such as virtual reality environments, where an action could be picked up from a sign but brought into a new environment. The main aim here being that the person who is interacting can act on the world, and gain an understanding, through enaction, of what makes a difference in the world, what actions really matter with regard to a specific science idea.

Second, the findings suggest that science exhibit designers or educators can benefit from drawing on a social sensorimotor perspective. This may relate to designing specific collaborative sensorial prompts in museums, such as boards demonstrating different types of collaborative action, such as in the case of Benjamin and his grandmother. These prompts may also give suggestions for collaboratively exploring movement and different body positions around the exhibit whilst interaction, to make sensorially hidden elements gradually perceptible. Visitors can be prompted to explore how the effects of two or more people doing the same action with an exhibit (where possible) changes the outcome and encourage subtle shifts in feedback to action being recognized and explored sensorially. To explore the limits of experiences with objects and exhibits in this way, together with others, can foster collaborative action around science.

Third, as shown through the interview data, children's gestural communication can demonstrate detailed ways in which science has been experienced. This suggests that children/families would benefit from opportunities to re‐enact aspects of their experiences after interaction with science‐themed activities. A chair or a corner of a room together with written prompts to engage in gesture and action‐based dialogs could complement this. As in the case of Benjamin and Matthew, this could give children an opportunity to present what they have noticed, what they discovered. It is not often that we are encouraged, socially, to attend to the details of our gestural communication. We are not regularly asked to reflect upon, or ask deepening questions about, what a gesture meant exactly, or if someone could “show that again.” Gestures can, however, as this study (and others) have demonstrated (e.g., Goldin‐Meadow, 2015; Thomas Jha et al., 2021), reveal much detail about different individuals’ experience. Although families attend to their children's communication, and young children are known to rely much on bodily forms of communication, the benefits of carving space for noticing and attending to gestural communication are not well recognized. Museums may address this through affording a comfortable corner to share moments of re‐enactment storytelling—what the child, and the adult, really noticed at the exhibit.

Although designing for learning experiences from an enactive cognition perspective can be beneficial, this approach does not come without limitations. Considering enactive opportunities, and different opportunities, to sensorially accessing and having experiences with science can be time‐consuming for pedagogues. This approach suggests that it takes time and patience to attend to the details of the objects, and the types of experiences that they can afford. The work presented in this paper suggests future research would benefit from further understanding the qualities of sensorial experiences that are afforded by science‐themed objects foregrounding action—what types of enactive potentialities tend to be directly accessible, what types of dimensions require more “probing” enactively over time, and what, in collaborative action, makes experiencing science meaningful from an affective engagement perspective. These threads of research can open up new possibilities to design experiences that foreground access to and experiencing with science.

## 6 CONCLUSION

This paper builds on prior work to extend our understanding of the “enactive” role of intergenerational interactions, specifically in terms of embodied scaffolding, that fosters young children's experiences with science. Taking an exploratory microinteractional approach to the analysis, we drew on enactive cognition to analyze and conceptualize specific moment‐to‐moment processes of adult‐child actions during informal science meaning making experiences, the gestures and actions observed in subsequent interviews, and the relationship between the two. The findings show how the adult's sensorimotor role is significant in renewing possibilities for children's further relevant enacted experiences and in understanding the material environment to deepen scientific ideas—through revealing enactive potentialities within the environment. Given that young children in particular can benefit from sensorimotor experiences and embodied forms of communication that go beyond verbal exchanges around science, this paper has demonstrated the value in considering the enactive potentialities of objects and environments that give access to experiences with science ideas, and how intergenerational interaction can be supported through design of interactive science spaces to foreground enactive experiences.

Although a microinteractional analytical approach provides detailed insight into enactive processes, it necessarily draws on a small data set to illustrate the findings. This points to the need for further research to extend our understanding of (intergenerational) embodied scaffolding, not only in museum spaces with larger family groups, but also in other informal, or even formal, science learning contexts.

## CONFLICT OF INTEREST STATEMENT

The authors declare no conflicts of interest.

## AUTHOR CONTRIBUTIONS

**Minna O. Nygren**: Formal analysis; data curation; writing—original draft preparation; writing—review and editing. **Sara Price**: Conceptualization; methodology; supervision; funding acquisition; writing—review and editing. **Rhiannon Thomas Jha**: Conceptualization; methodology investigation; formal analysis; writing—review.

## ETHICS STATEMENT

Ethical approval was obtained through University College London ethics committee (approval number REC 957) before undertaking the research. Informed consent of all participants was obtained.

## Data Availability

Data are not available to share due to ethical and confidentiality reasons. The participants of this study did not give written consent for their data to be shared publicly. Thus, due to the sensitive nature of the research, supporting data are not available.
